# Evaluating large language models for domain-specific scholarly metadata classification

**DOI:** 10.3389/frma.2026.1882130

**Published:** 2026-09-16

**Authors:** Muhammad Haris, Maryam Badar

**Affiliations:** 1 TIB—Leibniz Information Center for Science and Technology, Leibniz University Hannover, Hannover, Germany; 2 L3S Research Center, Leibniz Information Center for Science and Technology, Leibniz University Hannover, Hannover, Germany

**Keywords:** large language models, prompt engineering, scholarly metadata classification, simulation and modeling research, text classification

## Abstract

**Introduction:**

Large language models (LLMs) have been widely applied to text classification; however, their effectiveness in domain-specific scholarly metadata classification remains insufficiently explored. This study investigates the use of open-source LLMs to classify modeling and simulation research articles across three predefined metadata dimensions: Industry, Application Area, and Modeling Method.

**Methods:**

Using a curated dataset of research articles from the AnyLogic research repository, we evaluated models from the Qwen2.5, Llama3.1, Mistral, Ministral, and Gemma3 families. We compared Direct, Few-shot, and Chain-of-Thought prompting using either the article title alone or the title and abstract together. Performance was evaluated using Macro-F1 across the three metadata dimensions.

**Results:**

The best overall configuration, Ministral-3-14B with Direct prompting and the title and abstract as input, achieved a Macro-F1 of 53.8%. Performance varied substantially across the metadata dimensions. Modeling Method was the easiest to classify, reaching a maximum Macro-F1 of 69.1% with Ministral-3–14B. The best Macro-F1 scores for Industry and Application Area were 57.6% and 39.4%, respectively. Including abstracts consistently improved classification performance across the evaluated models, whereas differences among the three prompting strategies were relatively small. Categories that were semantically overlapping, broadly defined, highly imbalanced, or not explicitly mentioned in the article text were particularly difficult to classify.

**Discussion:**

The findings indicate that open-source LLMs can support domain-specific scholarly metadata classification without task-specific fine-tuning. However, their moderate and dimension-dependent performance limits their suitability for fully automated fine-grained metadata enrichment. Richer contextual information, more clearly defined taxonomies, and human validation are therefore needed to improve the reliability of LLM-assisted scholarly metadata classification.

## 1 Introduction

The emergence of large language models (LLMs) has driven substantial advances across numerous domains, including computer science, life sciences, and engineering (Raiaan et al., 2024). LLMs have demonstrated strong capabilities in tasks such as information extraction (Dagdelen et al., 2024), labeling entities (Wang et al., 2025), and summarization (Keya et al., 2024), with growing applications in scholarly knowledge organization. However, their effectiveness for specialized, fine-grained classification tasks in technical domains remains underexplored, particularly in zero-shot and few-shot settings that do not rely on task-specific training data.

The growth of scientific articles has created significant challenges for scholarly information access and discovery (Bornmann and Mutz, 2015). Digital libraries and domain-specific repositories must support the classification and retrieval of research articles at the required level of granularity. Traditional bibliographic metadata, such as titles, authors, publication venues, and keywords, supports basic retrieval but often fails to capture the multiple dimensions of a research article, including its application domain, operational context, and methodological approach. This limitation is particularly evident in interdisciplinary and application-oriented fields, where an article may be relevant across several metadata dimensions yet remain discoverable through only a single broad category.

In the modeling and simulation domain (Borshchev and Filippov, 2004), this challenge is particularly significant because research articles often span multiple application and methodological dimensions. Simulation studies address diverse industries, such as healthcare, manufacturing, and logistics; examine operational problems, including supply chain management, process optimization, and strategic planning; and employ different modeling paradigms, such as Discrete-event modeling, agent-based modeling, and system dynamics. For example, an article on agent-based simulation of hospital emergency department operations may be relevant to healthcare practitioners, simulation researchers, and operations researchers for different reasons. Representing such an article through only one broad category may obscure other important aspects of its contribution. Multi-dimensional metadata describing the industry, application area, and modeling method can therefore support more precise faceted search and improve the discovery of relevant research.

Creating metadata manually is time-consuming, costly, and difficult to scale. Curators must understand both the technical methodology and the application context of each article before assigning appropriate labels. Instruction-tuned LLMs offer a promising alternative because they can perform classification in zero-shot and few-shot settings without task-specific fine-tuning (Brown et al., 2020). This flexibility is particularly useful when labeled data is limited or category definitions evolve over time. However, it remains unclear how reliably lightweight LLMs can perform fine-grained classification in a specialized technical domain, how their performance differs across metadata dimensions, and how prompt design and available textual context affect classification quality. Addressing these questions helps determine whether such models can support practical scholarly metadata classification tasks.

We address these questions by systematically evaluating multiple open-source LLMs for classifying Modeling and Simulation research articles across three metadata dimensions: industry, application area, and modeling method. Using a curated dataset of M&S articles, we compare three prompting strategies: direct, few-shot, and chain-of-thought prompting. The evaluation includes models from the Qwen2.5, Llama3.1, Mistral, Ministral, and Gemma3 families and uses either the article title alone or both the title and abstract as input to assess the effect of additional contextual information on classification performance.

Our contributions are as follows:

- We construct a curated, domain-specific dataset of 200 Modeling and Simulation research articles with repository-provided metadata labels for industry, application area, and modeling method. The dataset provides a structured evaluation resource for automated scholarly metadata classification in a specialized scientific domain.
- We present a systematic evaluation of open-source generative LLMs from the Qwen2.5, Llama3.1, Mistral, Ministral, and Gemma3 families for fine-grained scholarly metadata classification. The models are evaluated under Direct, Few-shot, and Chain-of-Thought prompting using either the article title alone or the title and abstract together. To contextualize their performance, we compare the generative LLMs with a supervised TF–IDF–SVM classifier, a zero-shot natural language inference baseline based on BART-large-MNLI, and a fine-tuned SciBERT classifier.
- We analyze how classification performance varies across model families, metadata dimensions, prompting strategies, and input configurations. The results provide insight into the effects of additional textual context, category granularity, semantic overlap, and class imbalance on classification difficulty.

The remainder of this article is organized as follows. Section 2 reviews related work on scholarly article classification and LLM-based text classification. Section 3 describes the methodology, including dataset construction, the classification setup, and prompt engineering. Section 4 presents the experimental results across models and discusses the main findings, as well as the study's limitations and future research directions. Finally, Section 5 concludes the article.

## 2 Related work

Although LLMs demonstrate reliable general-purpose capabilities, their effectiveness in specialized domain-specific tasks remains an active area of research. We organize the related work into three areas: scholarly document classification, metadata extraction, and prompt-engineering methods for classification.

### 2.1 Scholarly document classification

Recent studies have explored different ways of improving scholarly document classification with prompting, retrieval, data augmentation, and model combination. Wang et al. (2022) proposed a unified prompt-tuning approach that transfers knowledge across classification tasks, while Milios et al. (2023) addressed in-context learning with large label spaces through retrieval-augmented methods. Such retrieval-based approaches are particularly relevant to fine-grained classification tasks involving extensive taxonomies. Similarly, Balkus and Yan (2024) showed that LLM-generated data augmentation can improve short-text classification, indicating that synthetic examples may be useful when only limited textual context or labeled data are available. Classification becomes more challenging when it involves thousands of evolving taxonomy labels that cannot be included in a single LLM input. To address this issue, Tabatabaei et al. (2025) proposed a hybrid approach that combines LLMs with dense retrieval for zero-shot label assignment. Evaluated on scientific documents from SSRN, the method reduces the need for retraining when the underlying taxonomy changes and provides a flexible solution for classification under large and dynamic label spaces. Platt and Platt (2023) evaluated the effectiveness of generative artificial intelligence for scientific content analysis, including the automated identification of research methods from scientific documents. Their results demonstrate the potential of general-purpose generative models for analyzing scientific content, and provide an important baseline for LLM-based scholarly document analysis.

Other work has focused on improving classification through interaction and iterative refinement. Yu et al. (2024) proposed an interactive LLM-based framework in which the model generates category descriptions and refines representative keywords based on user feedback. Requiring only limited initial input, the unsupervised approach achieves notable improvements on benchmark datasets after a small number of feedback iterations.

Beyond individual-model prompting, different ensemble strategies have also been investigated. Zhang et al. (2025) introduced RGPT, an adaptive boosting framework that recurrently combines fine-tuned LLMs by adjusting the distribution of training samples and incorporating historical predictions. Across four benchmark datasets, RGPT outperformed eight state-of-the-art pre-trained language models and seven LLM-based approaches by an average of 2.90%, demonstrating the potential of ensemble methods to improve text classification beyond the performance of individual models. Huang and He (2025) reformulated text clustering as a classification task using LLM-based in-context learning, thereby avoiding the need for fine-tuned embedding models. Their two-stage framework first generates and consolidates candidate labels and then assigns the resulting labels to individual samples. The method achieves performance comparable to state-of-the-art embedding-based approaches while reducing the computational requirements associated with model fine-tuning.

### 2.2 Metadata extraction

Recent studies have examined how LLMs can support metadata extraction, completion, and standardization across different scholarly domains. Watanabe et al. (2024) investigated the ability of LLMs to extract research metadata from scholarly articles through a combination of quantitative and qualitative analysis. Although the models demonstrated broad extraction capabilities, their accuracy remained inconsistent, particularly when identifying dataset names and linking related information across publications. Building on this work, Alyafeai et al. (2025) introduced MOLE, a framework for automatically extracting metadata attributes from scientific articles that describe datasets. The framework uses a schema-driven extraction process supported by validation mechanisms and is evaluated across multiple input formats, thus demonstrating promising performance for structured metadata extraction. Similarly, Marini et al. (2025) developed Data Gatherer, an LLM-based system for identifying and extracting dataset references from scientific publications. Evaluated on two curated benchmark datasets, the system achieved high precision and recall.

Other studies have focused on metadata completion and standardization. Song et al. (2024) investigated LLM-based methods for completing missing metadata fields in physical sample records from museums and scientific repositories. Their approach combines record summarization, bottom-up traversal of hierarchical taxonomies, and Few-shot prompting, demonstrating the potential of zero-shot and Few-shot methods for specialized metadata-completion tasks in the Earth sciences. In the biomedical domain, Sundaram et al. (2025) examined the ability of GPT-4 to improve metadata standardization using 200 BioSample records related to lung cancer research. Although GPT-4 alone produced only marginal improvements, its integration with structured domain knowledge through CEDAR metadata templates resulted in statistically significant gains. Beyond extraction and completion, LLMs have also been applied to metadata transformation and interoperability. Morejón et al. (2025) addressed the alignment of domain-specific TEI metadata from the digital humanities with FAIR-compliant DCAT catalogs. Their LLM-assisted transformation approach generates and enriches metadata during the conversion from TEI to DCAT that supports the integration of domain-specific literary standards with cross-domain catalog infrastructures and improves metadata interoperability and discoverability.

### 2.3 Prompt engineering for classification tasks

Wang et al. (2023) proposed Self-Instruct, a method that improves LLM instruction-following capabilities by bootstrapping from self-generated examples. The approach achieved a 33% improvement on the Super-Natural Instructions benchmark without relying on human annotations. Liu X. et al. (2022) showed that continuous prompt tuning can achieve performance comparable to full fine-tuning while requiring fewer computational resources. In addition, Liu J. et al. (2022) examined the factors that influence the quality of in-context examples and provided practical guidance for few-shot prompt design. Han et al. (2022) demonstrated that hybrid approaches combining prompting with explicit rules can improve classification performance by incorporating domain knowledge.

## 3 Methodology

In this section, we present the methodology for classifying domain-specific scholarly articles into predefined categories. Specifically, we evaluate open-source large language models (LLMs) and compare them with three baselines: a supervised TF-IDF-SVM model, a zero-shot natural language inference model, and a fine-tuned SciBERT model. These approaches are evaluated for classifying Modeling and Simulation (M&S) articles across three metadata dimensions: industry, application area, and modeling method. Figure 1 illustrates the overall methodology, which comprises dataset construction, input and prompt design, LLM-based classification and labels prediction.

**Figure 1:**
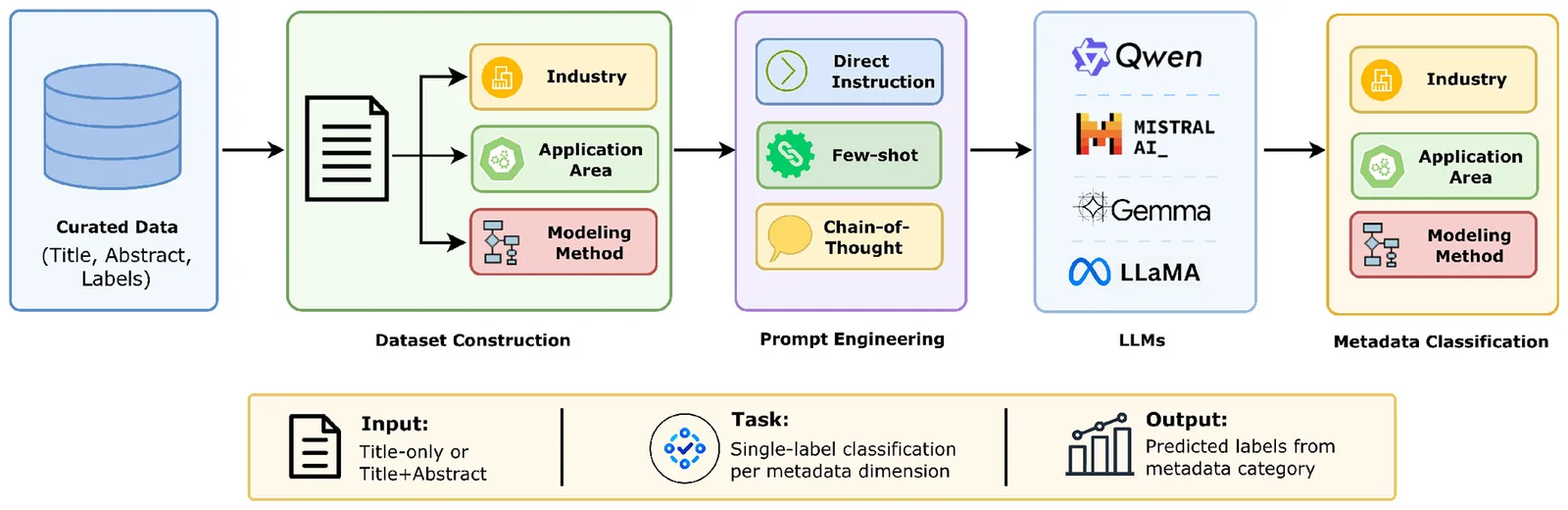
Proposed methodology comprising five stages: (1) collecting research article metadata from the AnyLogic repository; (2) constructing the dataset using article titles, abstracts and domain-specific metadata labels for industry, application area, and modeling method; (3) applying direct, few-shot and chain-of-thought prompting strategies; (4) using multiple LLMs to classify each article across the three metadata dimensions; and (5) parsing and validating the LLM responses to obtain the predicted labels.

### 3.1 Problem formulation

Let *D* denote a corpus containing *n* scholarly articles. Each article *d*_*i*_ is represented by its title *t*_*i*_ and abstract *a*_*i*_. It is assigned three metadata labels: industry *I*_*i*_, application area *A*_*i*_, and modeling method *M*_*i*_. The sets of possible labels for these dimensions are denoted by I, A, and M, respectively. Each article receives exactly one label from each set. Thus, *I*_*i*_ belongs to I, *A*_*i*_ belongs to A, and *M*_*i*_ belongs to M.

The three dimensions are classified independently. For each dimension, the classification system receives either the title alone or the title and abstract together and predicts one label from the corresponding label set. For generative LLMs, a dimension-specific prompt containing the task instructions and candidate labels is also provided. The predicted industry, application area, and modeling method labels are denoted by Î_*i*_, Â_*i*_, and ${\hat{M}}_{i}$, respectively. The study, therefore, addresses three related single-label, multi-class classification tasks rather than one task over all possible combinations of labels.

### 3.2 Dataset construction

We construct the dataset from Modeling and Simulation (M&S) research articles available in the publicly accessible AnyLogic research repository. The repository contains case studies, conference and journal articles describing a wide range of simulation and modeling applications. For each article, we collect the title, abstract, and repository-provided metadata labels for three dimensions: Industry, Application Area, and Modeling Method. Rather than independently re-annotating the articles, we retain these repository assignments as reference labels for the classification experiments.

In total, the dataset comprises 200 articles, each containing all required textual fields and associated metadata labels. Articles with missing titles, abstracts, or metadata assignments are excluded during dataset construction. Because the labels are provided directly by the repository, no independent multi-annotator annotation process is conducted, and inter-annotator agreement measures such as Cohen's kappa are therefore not applicable. The repository-provided assignments serve as the reference labels for evaluating the classification performance of the investigated models. Table 1 presents the distribution across all categories, while Table 2 provides representative examples from the constructed dataset. Each article is assigned exactly one label within each metadata dimension.

**Table 1:** Distribution of class labels across the three metadata classification dimensions.

Industry			Application area			Modeling method		
Label	*N*	%	Label	*N*	%	Label	*N*	%
Logistics	62	31.0	Supply chain management	36	18.0	Multimethod modeling	70	35.0
Healthcare	38	19.0	Manufacturing processes	28	14.0	Agent-based modeling	61	30.5
Industrial products	19	9.5	Management	26	13.0	Discrete-event modeling	49	24.5
Technology	17	8.5	Business processes	23	11.5	System dynamics modeling	20	10.0
Social research	16	8.0	Epidemiology simulation	17	8.5			
Consumer products	13	6.5	Strategic management	14	7.0			
Construction	10	5.0	Freight traffic modeling	14	7.0			
Energy systems	9	4.5	Asset management	10	5.0			
Science & education	6	3.0	Machine learning	6	3.0			
Aerospace	5	2.5	Personnel	6	3.0			
Military, defense, safety	5	2.5	Communication networks	5	2.5			
			Marketing	5	2.5			
			Labor market	5	2.5			
			Market modeling	5	2.5			
**Total**	**200**	**100.0**	**Total**	**200**	**100.0**	**Total**	**200**	**100.0**

Percentages are calculated over the 200 articles in the dataset.

**Table 2:** Sample annotated instances from the dataset.

Title (shortened)	Industry	Application area	Method
Sttar: a simheuristics-enabled scheme for multi-stakeholder coordination of aircraft turnaround operations	Aerospace	Strategic management	Multimethod modeling
Bloodchainsim—a simulation environment to evaluate digital innovations in blood supply chains	Healthcare	Supply chain management	Multimethod modeling
An agent-based simulation of a tuberculosis epidemic: understanding the timing of transmission	Healthcare	Epidemiology simulation	Agent-based modeling
Evaluation of agent-based and discrete-event simulation for modeling construction earthmoving operations	Construction	Management	Multimethod modeling
A simulation-based approach for line balancing under demand uncertainty in a production environment	Industrial products	Manufacturing processes	Discrete-event modeling
Using simulation modeling for IT cost analysis	Technology	Business processes	System dynamics modeling
Multimethod modeling and simulation of a vertical lift module with an integrated buffer system	Consumer products	Asset management	Multimethod modeling
Hybrid simulation-based optimization for second-generation biofuel supply chain network design	Logistics	Supply chain management	Agent-based modeling

Each instance consists of a article title and abstract annotated with industry, application area, and simulation method labels.

The three metadata dimensions capture distinct aspects of M&S research articles and support their classification, search, and filtering. *Industry* enables domain-based filtering, for example, identifying articles related to healthcare. *Application Area* supports functional searches, such as locating studies on supply chain optimization, whereas *Modeling Method* facilitates methodological queries, such as identifying agent-based approaches to a particular problem. Accurate classification across these dimensions can further support faceted search interfaces, in which researchers combine multiple filters, for example, to retrieve Discrete-event simulation studies of healthcare operations.

These metadata dimensions describe different aspects of an article, which can make classification difficult. For example, articles from different Industry categories, such as *Logistics, Healthcare*, and *Industrial Products*, can address the same Application Area, such as *Supply Chain Management*. Industry indicates the broader domain in which the study is conducted, whereas Application Area describes the specific problem or activity being investigated. The classification task is particularly challenging because articles may use domain-specific terms instead of the exact category names. For instance, an article about optimizing patient flow should be classified under the *Healthcare* Industry even if the word “Healthcare” does not appear in its title or abstract. These differences in meaning and terminology make classification across the three metadata dimensions particularly challenging.

### 3.3 Category definitions

Our classification schema comprises three metadata dimensions, each capturing a distinct aspect of Modeling and Simulation research.

#### 3.3.1 Industry

Industry refers to the primary domain in which the simulation research is applied. Examples include *Healthcare*, such as patient flow optimization and resource allocation; *Manufacturing*, such as production line design and quality control; and *Logistics*, such as warehouse operations and delivery networks. Industry categories represent the target application domain rather than the methodological approach used in the study. For example, an article titled “*Emergency Department Staffing Optimization Using Discrete-event Simulation”* is classified under the *Healthcare* Industry category.

#### 3.3.2 Application area

Application area refers to the specific operational context or functional problem addressed by the simulation. For example, *Supply Chain Management* concerns procurement and distribution networks; *Healthcare Operations* addresses clinical workflows and capacity planning; and *Manufacturing Processes* covers manufacturing-system simulation and production scheduling. Application Area categories are generally more specific than Industry categories and may span multiple domains. For instance, *Supply Chain Management* may be relevant to studies in Manufacturing, Logistics, or Healthcare.

#### 3.3.3 Modeling method

Modeling Method refers to the fundamental simulation paradigm employed in the study. *Discrete-event Modeling* represents systems as sequences of events occurring over time, such as queuing systems and process flows. *Agent-Based Modeling* represents systems through autonomous entities that follow predefined decision rules, such as pedestrians or market participants. *System Dynamics* represents system behavior through stocks, flows, feedback relationships, and equations that describe how the system changes over time, for example, in studies of epidemic spread or resource depletion. *Multimethod Modeling* combines two or more simulation approaches, for example, an integrated supply-chain model that represents customer behavior through agent-based modeling and warehouse operations through Discrete-event modeling.

### 3.4 Article classification

We evaluate open-source instruction-tuned large language models (LLMs) and three baseline approaches for classifying scholarly articles into predefined metadata categories. The generative models belong to the Qwen2.5, Llama3.1, Mistral, Ministral, and Gemma3 families. Each LLM is evaluated under Direct, Few-shot, and Chain-of-Thought prompting using either the article title alone or the title and abstract together. For comparison, we evaluate a supervised TF–IDF–SVM classifier, a zero-shot natural language inference baseline based on BART-large-MNLI, and a fine-tuned SciBERT classifier. Each system classifies articles independently across three metadata dimensions: Industry, Application Area, and Modeling Method.

### 3.5 Baseline models

To provide a comparison with generative language models, we implement three complementary baselines: a traditional TF–IDF–SVM classifier, a zero-shot natural-language-inference model, and an existing SciBERT encoder. These baselines represent three different levels of supervision. TF–IDF–SVM learns task-specific classifiers from the labeled corpus using sparse lexical features; BART-large-MNLI performs classification without training on the study dataset; and SciBERT is fine-tuned on the labeled corpus through cross-validation. All approaches are evaluated independently for industry, application area, and modeling method.

#### 3.5.1 TF–IDF–SVM

For the traditional supervised baseline, we combine term frequency–inverse document frequency (TF–IDF) representations with a linear support vector machine. Two input configurations are evaluated: the title alone and the concatenation of the title and abstract. Text is normalized through lower casing, Unicode accent stripping, and English stop-word removal. The TF–IDF representation includes unigrams and bigrams, uses sublinear term-frequency scaling, and excludes terms occurring in fewer than two documents. Classification is performed using LinearSVC. The TF–IDF vectorizer and the SVM classifier are encapsulated in a single pipeline. The primary evaluation uses stratified five-fold cross-validation. In each round, approximately 80% of the articles are used for training and 20% for testing, such that every article receives exactly one out-of-fold prediction.

#### 3.5.2 Zero-shot NLI baseline

As a non-generative zero-shot baseline, we employ BART-large-MNLI (facebook/bart-large-mnli). Metadata classification is formulated as a natural-language-inference task in which the title or title and abstract serve as the input. Each candidate category is inserted into a fixed task-specific hypothesis template:

- *The primary industry discussed in this article is [CATEGORY]*.
- *The primary application area of this article is [CATEGORY]*.
- *The primary modeling method used in this article is [CATEGORY]*.

For every article, BART-large-MNLI evaluates the hypotheses corresponding to all categories within the relevant metadata dimension. Because the task is defined as single-label classification, we normalize the candidate scores jointly by setting multi_label=False, and the category receiving the highest score is selected as the prediction.

#### 3.5.3 Fine-tuned SciBERT baseline

To evaluate a supervised, domain-specific language encoder, we fine-tune SciBERT using the allenai/scibert_scivocab_uncased checkpoint. We select SciBERT because it is pretrained on scientific publications and therefore provides representations adapted to scholarly language. Separate classifiers are trained for industry, application area, and modeling method. We evaluate SciBERT using stratified five-fold cross-validation. Fine-tuning uses AdamW with a learning rate of 2 × 10^−5^, a weight decay of 0.01, a linear learning-rate schedule, and a warm-up ratio of 0.1. The models are trained for five epochs, with gradient norms clipped to 1.0. To account for the imbalanced category distribution, inverse-frequency class weights derived from each training fold are incorporated into the cross-entropy loss. Input sequences are limited to 512 tokens, and dynamic batch-level padding is used to reduce unnecessary computation.

### 3.6 LLM-based classification

We evaluate instruction-tuned LLMs using prompts designed specifically for the metadata classification task. For each article, separate prompts are instantiated for industry, application area, and modeling method classification. Each prompt contains the article title and abstract, the predefined category list for the corresponding metadata dimension, and an instruction to select exactly one category. The Few-shot prompts include labeled examples to demonstrate the expected relationship between article content and category labels. Figure 2 presents the chain-of-thought prompt template, which guides the model through a structured consideration of the article topic, relevant textual indicators, and the available categories before producing its prediction.

**Figure 2:**
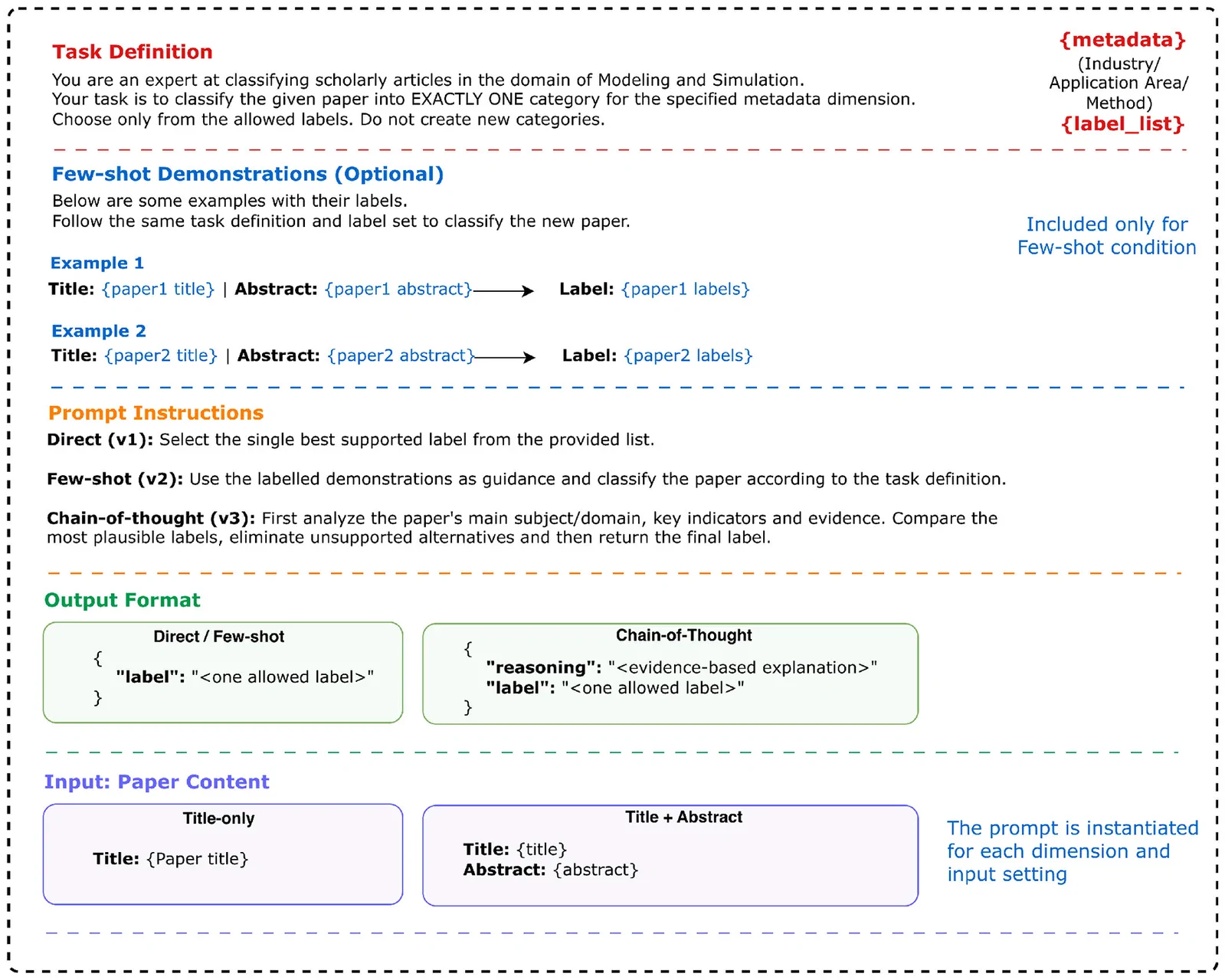
Unified prompt template used for scholarly metadata classification. The prompt comprises four components: task definition, optional few-shot demonstrations, strategy-specific instructions, and output format. It is instantiated separately for industry, application area, and modeling method, resulting in three independent prompts per article. Depending on the experimental setting, the input consists of either the article title alone or both the title and abstract, and the prompting strategy is direct, few-shot, or chain-of-thought. Highlighted placeholders indicate the fields adapted for each metadata dimension.

As shown in the figure, the chain-of-thought template instructs the model to reason step by step before selecting a category, an approach that can improve performance on tasks requiring intermediate reasoning (Wei et al., 2022). Placeholders enclosed in curly braces allow the template to be instantiated dynamically for different metadata dimensions, category lists, and article instances. For few-shot prompting, labeled examples are included for each metadata dimension. We prepare a separate demonstration set that remains fixed across all target articles and evaluated models. The examples are selected to illustrate the expected input–output format and to represent different labels within the corresponding metadata dimension. They are not selected dynamically based on semantic similarity to the target article. Using a fixed demonstration set ensures that all models are evaluated under the same prompting conditions.

For each article, the three metadata dimensions are classified independently. Separate LLM inference calls are performed for industry, application area, and modeling method, with the corresponding category list included in each prompt. Each article, therefore, produces three classification outputs, one for each metadata dimension, and no joint multi-field prompt is used. The predictions for the three metadata dimensions are evaluated independently against their corresponding reference labels.

#### 3.6.1 Inference settings

All models are executed locally through Ollama using deterministic settings. The temperature is set to 0.0, the random seed to 42, and the context-window size to 8,192 tokens. Streaming is disabled, and structured JSON output is enforced through a predefined output schema. For few-shot prompting, three labeled demonstrations are included in each prompt. Other decoding parameters, including top-*p*, top-*k*, and repetition penalties, are not explicitly modified and therefore follow the defaults of the corresponding Ollama model configuration.

#### 3.6.2 LLM output parsing and validation

The LLM responses are requested in a structured JSON format containing a predicted label and, for chain-of-thought prompting, a reasoning field. The output schema restricts the prediction to the predefined labels of the corresponding metadata dimension. Once LLM generates the response, it is parsed, and the value of the label field is extracted. The extracted label is normalized and compared with the predefined labels using case-insensitive matching. Predictions that cannot be mapped unambiguously to an allowed label are marked as invalid. Responses that cannot be parsed or do not contain the required label field are recorded as processing errors. The same parsing and validation procedure is applied consistently across all models, prompt strategies, and input configurations.

## 4 Results & discussion

This section presents the classification performance of the evaluated open-source LLMs across the three metadata dimensions. We compare the models using three prompting strategies—direct, few-shot, and chain-of-thought—and two input configurations: the article title alone and both the title and abstract. This experimental design allows us to assess the effects of model choice, prompting strategy, and additional textual context on classification performance. We first describe the evaluation metrics and then present the overall, input-specific, prompt-specific, and dimension-specific results.

### 4.1 Evaluation metrics

We evaluate classification performance using Precision, Recall and F1-score. Equations 1–4 show how the corresponding evaluation metrics are calculated. These metrics are computed separately for industry, application area, and modeling method. For a given class, Precision measures the proportion of instances predicted as that class that are correctly classified:


$$\begin{array}{l} \text{Precision}=\frac{TP}{TP+FP}, \end{array}$$ (1)

where *TP* denotes the number of true positives and *FP* denotes the number of false positives.

Recall measures the proportion of gold-standard instances of a class that are correctly identified:


$$\begin{array}{l} \text{Recall}=\frac{TP}{TP+FN}, \end{array}$$ (2)

where *FN* denotes the number of false negatives.

The F1-score is the harmonic mean of Precision and Recall:


$$\begin{array}{l} \text{F}1=2\times \frac{\text{Precision}\times \text{Recall}}{\text{Precision}+\text{Recall}}. \end{array}$$ (3)

Because all three metadata dimensions are multi-class classification tasks, we compute Precision, Recall, and F1-score independently for each class. We primarily report Macro-F1, which assigns equal importance to every class regardless of its frequency:


$$\begin{array}{l} \text{Macro}\text{-F}1=\frac{1}{C}\overset{C}{\underset{c=1}{\sum }}\text{F}1_{c}, \end{array}$$ (4)

where *C* denotes the number of classes in the corresponding metadata dimension.

Macro-F1 is particularly important for this dataset because the category distributions are highly imbalanced. It prevents frequent classes from dominating the reported performance and therefore provides a more balanced assessment across both frequent and rare categories.

For each model, prompt strategy, input configuration, and metadata dimension, the metrics are computed over all 200 articles. The overall Macro-F1 for a configuration is calculated as the arithmetic mean of the Macro-F1 scores for Industry, Application Area, and Modeling Method.

All experiments are executed on the same RTX 5090 GPU 32 GB environment using consistent prompt templates, inference settings, and evaluation scripts. Table 3 presents the best-performing prompt and input configuration for each evaluated model. Table 4 compares title-only and title + abstract input.

**Table 3:** Best-performing configuration for each evaluated baseline and language model.

Model	Approach/prompt	Best input	Industry	Application area	Method	Overall
Baselines						
TF–IDF–SVM	Supervised (5-fold CV)	Title + abstract	41.19	23.21	53.72	39.36
Zero-shot NLI	Zero-shot	Title + abstract	49.35	33.16	52.64	45.05
SciBERT	Fine-tuned (5-fold CV)	Title + abstract	26.9	23.7	33.5	28.0
Generative language models						
Ministral-3-14B	Direct	Title + abstract	55.9	36.3	**69.1**	**53.8**
Qwen2.5-14B	Chain-of-thought	Title + abstract	55.0	**39.4**	66.8	53.7
Qwen2.5-7B	Few-shot	Title + abstract	**57.6**	39.4	63.9	53.6
Gemma3-12B	Direct	Title + abstract	53.9	38.6	65.4	52.6
Mistral-7B	Few-shot	Title + abstract	47.6	38.4	62.0	49.3
Llama3.1-8B	Direct	Title + abstract	52.1	33.7	59.6	48.5
Qwen2.5-3B	Chain-of-thought	Title + abstract	53.6	26.4	51.4	43.8

TF–IDF–SVM results are based on pooled out-of-fold predictions under stratified five-fold cross-validation. Fine-tuned SciBERT is also evaluated using stratified five-fold cross-validation. Zero-shot NLI and the generative language models classify the complete gold dataset directly because they are not trained on the study dataset. All approaches are evaluated against the same final gold labels. Overall Macro-F1 is calculated as the unweighted arithmetic mean of the industry, application area, and modeling method Macro-F1 scores. Bold values indicate the highest performance achieved by any method for the corresponding evaluation metric.

**Table 4:** Comparison of the best prompt strategies for title-only and title-plus-abstract inputs.

Model	Best title prompt	Title	Best title + abstract prompt	Title + abstract	Difference
Gemma3-12B	Few-shot	45.2	Direct	52.6	**+7.4**
Llama3.1-8B	Few-shot	46.0	Direct	48.5	+2.5
Ministral-3-14B	Direct	**49.9**	Direct	**53.8**	+3.9
Mistral-7B	Few-shot	48.5	Few-shot	49.3	+0.8
Qwen2.5-14B	Few-shot	49.6	Chain-of-thought	53.7	+4.1
Qwen2.5-3B	Few-shot	41.8	Chain-of-thought	43.8	+2.0
Qwen2.5-7B	Few-shot	48.7	Few-shot	53.6	+5.0

Scores and differences are reported as overall macro-F1 percentages. Bold values indicate the highest performance achieved by any method for the corresponding evaluation metric.

### 4.2 Baseline evaluation

Using title and abstract input, TF–IDF–SVM achieves Macro-F1 41.19% for Industry, 23.21% for Application Area, and 53.72% for Modeling Method, resulting in an overall Macro-F1 of 39.36%. BART-large-MNLI obtains an overall Macro-F1 of 45.05% with title and abstract input, representing an improvement of 8.95% over title-only input and 5.69 points over TF–IDF–SVM. SciBERT results are low as compared to the other two baselines, and it requires a large amount of training data. Across all baselines, application area remains the most challenging classification dimension.

### 4.3 LLMs evaluation

Table 3 presents the best prompt and input configuration for each model based on the overall Macro-F1 across the three metadata dimensions. Among the generative language models, Ministral-3–14B achieves the highest overall Macro-F1 of 53.8% using Direct prompting with the article title and abstract as input. Qwen2.5–14B follows closely with 53.7% using Chain-of-Thought prompting, while Qwen2.5–7B achieves 53.6% using Few-shot prompting. Gemma3–12B obtains 52.6%, followed by Mistral–7B with 49.3%, Llama3.1–8B with 48.5% and Qwen2.5–3B with 43.8%. The best configuration for every model uses both the title and abstract, indicating that the additional context provided by the abstract generally improves overall classification performance.

Across the evaluated LLMs, the best title with abstract configuration consistently outperforms the best title-only configuration. As shown in Table 4, the best overall Macro-F1 for Qwen2.5–3B increases from 41.8% with title-only input to 43.8% with title-plus-abstract input, representing an improvement of 2.0 percentage points. The largest gain is observed for Gemma3–12B, whose overall Macro-F1 increases from 45.2% to 52.6%, an improvement of 7.4 percentage points. The smallest gain is observed for Mistral–7B, which improves from 48.5% to 49.3%, corresponding to 0.8 percentage points. These results indicate that abstracts generally provide useful contextual information that is unavailable or insufficiently expressed in titles, although the magnitude of their contribution varies across models and classification dimensions.

Overall, the amount of input information has a greater effect on aggregate performance than the choice of prompting strategy. The best title-plus-abstract configuration improves the overall Macro-F1 for every evaluated model, whereas the differences among direct, few-shot, and chain-of-thought prompting remain relatively small. Few-shot prompting does not consistently outperform Direct or chain-of-thought prompting, suggesting that a small number of demonstrations may not fully represent the diversity and imbalance of the classification categories.

Model size also influences performance, although larger models do not consistently outperform smaller alternatives. Ministral-3–14B achieves the best overall result and the highest scores for Industry and Modeling Method, whereas the smaller Mistral–7B obtains the highest application area Macro-F1. Qwen2.5–7B also performs competitively with Qwen2.5–14B overall, achieving 53.6% compared with 53.7%. These findings indicate that performance depends not only on model size but also on the interaction among the model, prompting strategy, input information, and classification dimension. Overall, the models perform most reliably on Modeling Method and least reliably on application area, where the categories are broader, imbalanced, and semantically overlapping.

### 4.4 Confusion-matrix analysis

Figures 3–5 show row-normalized confusion matrices for the configurations with the highest dimension-level Macro-F1 scores among those reported in Table 3. Rows represent gold labels, columns represent predicted labels, and each cell reports the percentage of instances from a gold class assigned to the corresponding predicted class.

**Figure 3:**
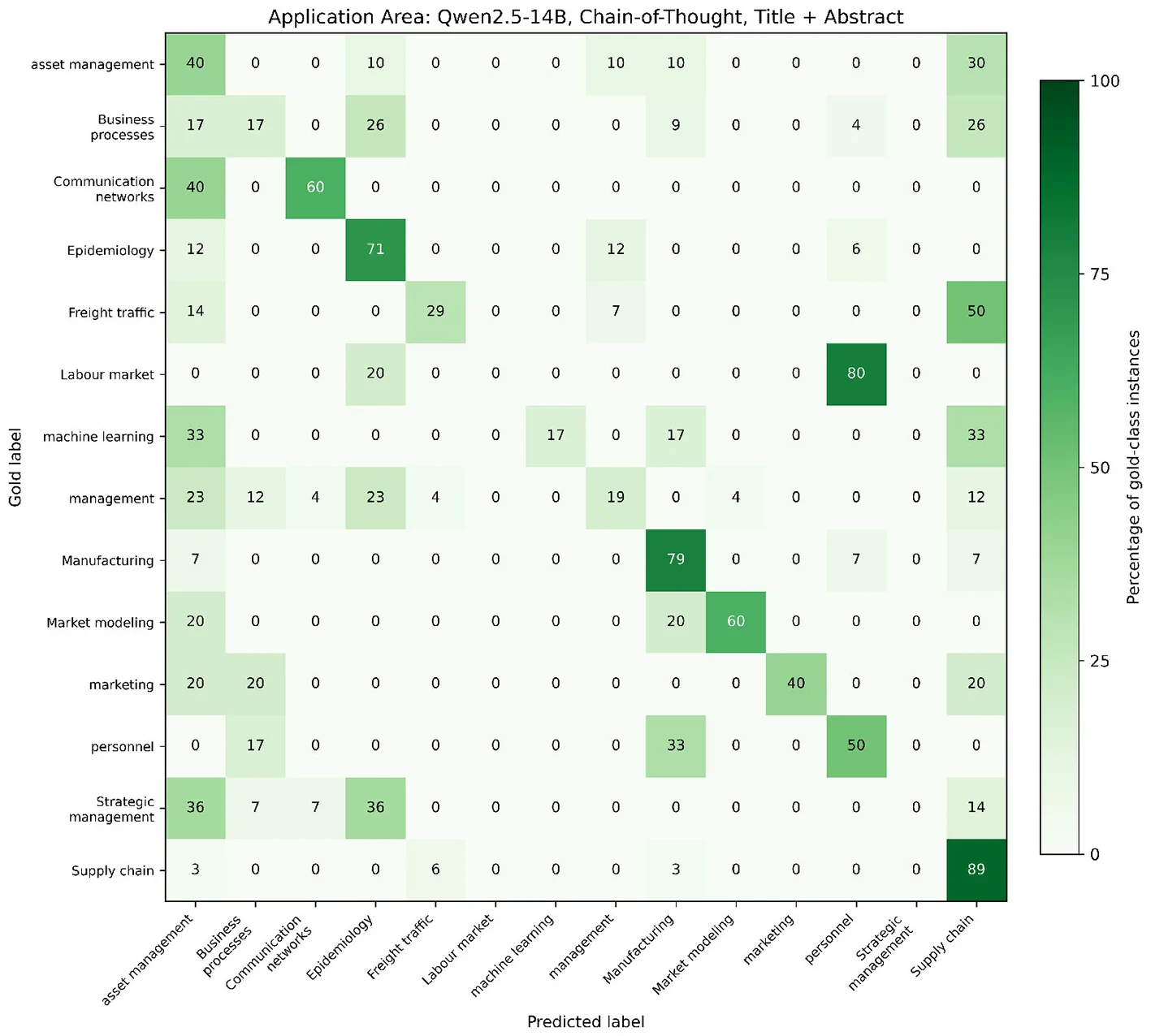
Row-normalized confusion matrix for application area classification using Qwen-2.5–14B with Direct prompting and chain-of-thought input, the configuration achieving the highest application area Macro-F1. Rows represent gold labels, columns represent predicted labels, and cell values indicate the percentage of gold-class instances assigned to each predicted class.

For application area classification (Figure 3), Qwen2.5–14B with chain-of-thought prompting and title with abstract input achieves its highest recall for Supply Chain Management at 89%, followed by manufacturing processes at 79%, epidemiology simulation at 71% and communication networks and market modeling at 60% each. Personnel achieves 50% recall, while asset management and marketing each achieve 40%. Lower recalls are observed for freight traffic modeling at 29%, management at 19%, and business processes and machine learning at 17% each. Labor market and strategic management receive 0% recall. The most prominent errors include 80% of labor market instances being classified as personnel, 50% of freight traffic modeling instances being classified as supply chain management, and 40% of communication networks instances being classified as asset management. Strategic management is primarily confused with asset management and epidemiology simulation, each accounting for 36% of its instances.

For industry classification (Figure 4), Qwen2.5–7B with few-shot prompting and title with abstract input achieves 100% recall for Aerospace, followed by healthcare at 95%, industrial products at 79%, energy systems at 78%, logistics at 77%, and construction at 70%. Consumer products achieves 54% recall, while military, defense, and safety, technology, and social research achieve 40%, 35%, and 25%, respectively. Science and education receives 0% recall, with its instances primarily classified as industrial products and technology at 33% each. Other notable errors include 60% of military, defense, and safety instances being classified as logistics and 46% of consumer products instances being divided equally between Industrial Products and Logistics.

**Figure 4:**
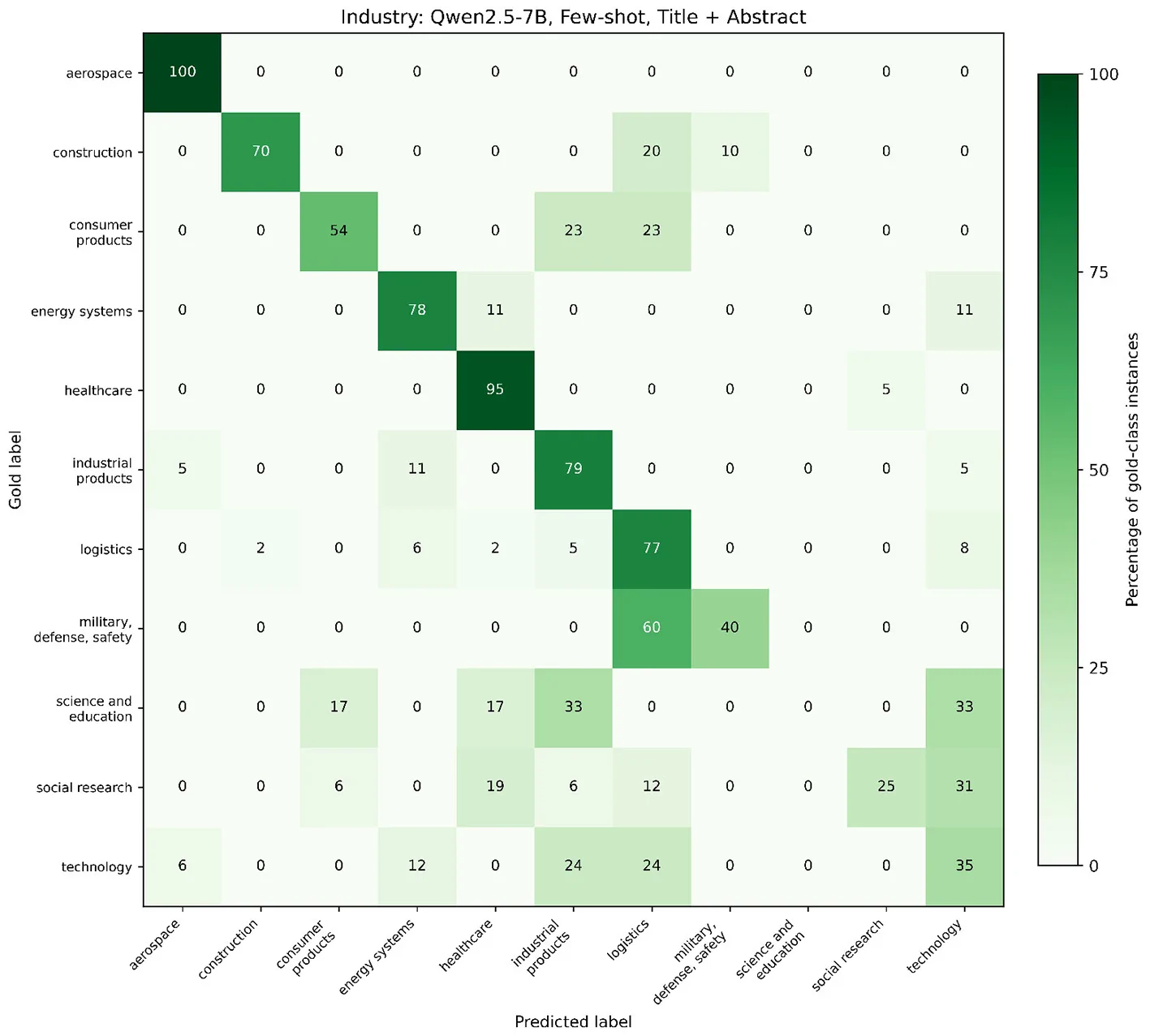
Row-normalized confusion matrix for industry classification using Qwen-2.5–7B with Direct prompting and title with abstract input.

The modeling method matrix (Figure 5) displays the clearest diagonal pattern, consistent with its stronger aggregate performance. Ministral-3–14B with Direct prompting and title-plus-abstract input achieves 79% recall for agent-based modeling, 76% for discrete-event modeling, 64% for multimethod modeling, and 55% for system dynamics modeling. Most errors occur between related paradigms: 24% of multimethod modeling instances are classified as discrete-event modeling, while 16% of discrete-event modeling instances are classified as multimethod modeling. System dynamics Modeling is primarily confused with discrete-event modeling at 25% and agent-based modeling at 15%. These patterns indicate that the modeling method is more reliably identified than the industry and application area, while errors concentrate among semantically related or overlapping labels.

**Figure 5:**
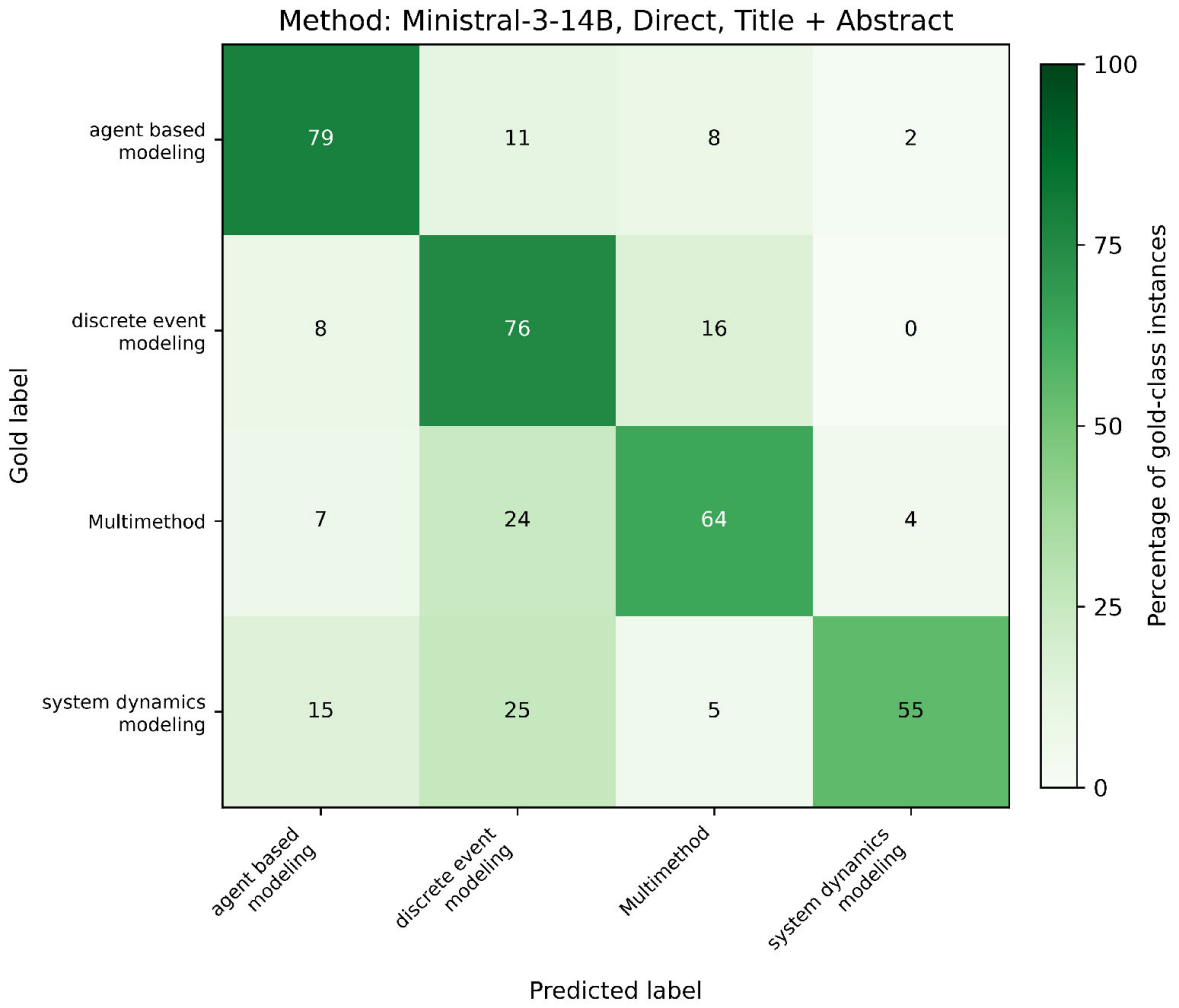
Row-normalized confusion matrix for modeling method classification using Ministral-3–14B with direct prompting and title with abstract input.

The results indicate that the effectiveness of LLM-based scholarly metadata classification depends on the selected model and also on the metadata dimension being predicted, the amount of contextual information provided, and the prompting strategy employed. These findings are particularly relevant to specialized scholarly communication settings, where classification requires interpreting domain-specific terminology and distinguishing between categories that may not be stated explicitly in article titles or abstracts.

Class imbalance also affects the interpretation of the results. Some categories contain many articles, whereas others contain only one or a few instances. Consequently, class-level Precision, Recall, and F1-scores for rare categories are highly sensitive to individual predictions. We have reported the Macro-F1 as a primary evaluation measure because it assigns equal importance to each class, but it can also be strongly affected by low-support categories.

Including the abstract consistently improves performance compared with using the title alone. All evaluated models achieve their best overall configuration with title and abstract input. This finding indicates that titles often provide insufficient information for identifying the study context, particularly for industry and application area. Abstracts provide additional evidence about the modeled system, research objective, operational problem, and methodological design, which supports more reliable classification.

For libraries, repositories, and scholarly information systems, a practical deployment strategy could combine LLM predictions with controlled vocabularies, category definitions, and human-in-the-loop verification. Such a workflow could reduce the effort required for manual metadata curation while maintaining appropriate quality control. The results further suggest that richer textual context should be provided whenever available, particularly for metadata dimensions that cannot be inferred directly from article titles.

### 4.5 Limitations

This study is limited by the relatively small dataset of 200 articles drawn from a single source, the AnyLogic research repository. As a commercial platform, AnyLogic may reflect particular modeling paradigms, application domains, taxonomy choices, and labeling conventions, which limit the generalizability of the findings to the broader modeling and simulation literature. The dataset is also highly imbalanced, with several categories represented by a few articles. The evaluation is restricted to open-source LLMs with up to 14 billion parameters and uses only article titles and abstracts, which may omit methodological and contextual information available in the full text. In addition, the study adopts a predefined single-label classification setting and evaluates a limited set of prompt formulations. Other prompting strategies may further improve classification performance.

### 4.6 Future directions

Future work could evaluate larger and more diverse language models, incorporate additional article content, such as the introduction, methodology, and model-description sections, and expand the dataset. Increasing the number of examples for rare categories would support more reliable class-level evaluation and improve dataset coverage. Further research could investigate multi-label classification, taxonomy-aware prompting, and contrastive category definitions.

## 5 Conclusion

This study evaluates open-source LLMs for classifying Modeling and Simulation research articles across three metadata dimensions: industry, application area, and modeling method. The experiments compare direct, few-shot, and chain-of-thought prompting using either the article title alone or both the title and abstract as input. Ministral-3–14B achieves the highest overall Macro-F1 score of 53.8%, using chain-of-thought prompting with both the title and abstract. Modeling method is the easiest dimension, reaching around 70.0% Macro-F1 with Ministral-3–14B, whereas the best industry and application area scores are 57.6% and 39.4%, respectively. Including abstracts consistently improves performance, while differences among prompting strategies remain relatively modest. The findings show that open-source LLMs can support scholarly metadata enrichment without task-specific fine-tuning, but performance remains sensitive to the specific class, the limited contextual information available, and semantic overlap among categories.

## Data Availability

The raw data supporting the conclusions of this article will be made available by the authors, without undue reservation.
